# Methylene blue promotes quiescence of rat neural progenitor cells

**DOI:** 10.3389/fncel.2014.00315

**Published:** 2014-10-07

**Authors:** Luokun Xie, Gourav R. Choudhury, Jixian Wang, Yong Park, Ran Liu, Fang Yuan, Chun-Li Zhang, Thomas Yorio, Kunlin Jin, Shao-Hua Yang

**Affiliations:** ^1^Department of Pharmacology and Neuroscience, University of North Texas Health Science CenterFort Worth, TX, USA; ^2^Department of Neurosurgery, Beijing Neurosurgical Institute, Beijing Tiantan Hospital, Capital Medical UniversityBeijing, China; ^3^Department of Molecular Biology, University of Texas Southwestern Medical CenterDallas, TX, USA

**Keywords:** methylene blue, neural progenitor cell, quiescence, proliferation, neurogenesis

## Abstract

Neural stem cell-based treatment holds a new therapeutic opportunity for neurodegenerative disorders. Here, we investigated the effect of methylene blue on proliferation and differentiation of rat neural progenitor cells (NPCs) both *in vitro* and *in vivo*. We found that methylene blue inhibited proliferation and promoted quiescence of NPCs *in vitro* without affecting committed neuronal differentiation. Consistently, intracerebroventricular infusion of methylene blue significantly inhibited NPC proliferation at the subventricular zone (SVZ). Methylene blue inhibited mTOR signaling along with down-regulation of cyclins in NPCs *in vitro* and *in vivo*. In summary, our study indicates that methylene blue may delay NPC senescence through enhancing NPCs quiescence.

## INTRODUCTION

Methylene blue is a heterocyclic aromatic chemical compound that is used to treat malaria (Dormoi et al., 2013), methemoglobinemia (Zenk, 2001), and ifosfamide neurotoxicity (Patel, 2006). Recent studies have identified its potential neuroprotective action in ischemic stroke, Parkinson’s disease (Wen et al., 2011), and Alzheimer’s disease (Congdon et al., 2012). In addition, MB modulates metabolism as an alternative mitochondrial electron carrier and enhances mitochondrial oxidative phosphorylation (Wen et al., 2011; Poteet et al., 2012, 2013). MB can induce autophagy to protect neurons from serum deprivation (Xie et al., 2013). Metabolism is known to play a pivotal role in dictating whether a cell proliferates, differentiates, or remains quiescent (Shyh-Chang et al., 2013). Coincidentally, MB has been demonstrated to delay fibroblast senescence (Atamna et al., 2008) and inhibit glioblastoma cells proliferation (Poteet et al., 2013). However, whether MB has effects on NPCs has not been addressed.

Neural progenitor cells reside in distinct regions of adult mammalian brains and have the capacity for self-renewal and giving rise to three neural lineages through asymmetric cell division (Gage, 2000). NPCs are involved in the maintenance of normal brain homeostasis and participate in CNS (central nervous system) regeneration and repair (Barkho and Zhao, 2011; Liu and Rando, 2011). Stem cell exhaustion has been recognized as one of the hallmarks of aging (López-Otín et al., 2013). Depletion of NPCs, possibly through loss of self-renewal capacity, has been indicated to be responsible for declining neurogenesis with age (Renault et al., 2009). Thus, maintenance of self-renewal and prevention of senescence is of great importance for longevity research and anti-aging therapeutics. In this study, we determined the effect of MB on the proliferation, self-renewal and differentiation of NPCs *in vitro* and *in vivo*. We demonstrated that MB inhibited proliferation and promoted quiescence of NPCs, but not affecting committed neuronal differentiation. In addition, we found that MB down-regulated mTOR and cyclins expression in NPCs.

## MATERIALS AND METHODS

### EXPERIMENTAL ANIMALS

All experiments were conducted in compliance with institutional guidelines and NIH Guidelines for the Use of Animals. All animal procedures were approved by the University of North Texas Health Science Center Animal Care and Use Committee. Male Sprague-Dawley rats weighing 250 – 300 g were purchased from the Jackson Laboratory. Two mM MB (American Regent, Shirley, NY, USA) in 100 μl of artificial cerebrospinal fluid was administered into the left lateral ventricle using Alzet osmotic minipumps (0.5 μl/h) for 7 days. Bromodeoxyuridine (50 mg/kg BrdU, Sigma-Aldrich, St. Louis, MO, USA) was intraperitoneally injected twice a day during the first 3 days of MB treatment. All rats were euthanized and brains were collected for cryosectioning.

### NEUROSPHERE CULTURE AND *IN VITRO* MB TREATMENT

Neurosphere cultures were performed following previous literature (Imura et al., 2003). Briefly, SVZ of mixed postnatal day 1 rats were digested with 0.25% Trypsin-EDTA (Invitrogen, Grand Island, NY, USA) at 37°C for 30 min. Cells were dissociated by trituration and resuspended at 50,000 cells/ml in Neurobasal medium (Invitrogen, Grand Island, NY, USA) supplemented with B27, 10 ng/ml fibroblast growth factor-2 (FGF-2) and 10 ng/ml epidermal growth factor (EGF, both from Peprotech, Rocky Hill, NJ, USA). Culture medium in the presence or absence of 5 μM MB was changed every 3 days. To assess the neurosphere formation in serial passage assay, primary neurospheres were mechanically dissociated into single cell suspension. 1 × 10^4^ NPCs in 1 ml of culture medium were incubated for 7 days in 12-well suspension culture plates. The number of neurospheres was counted at day 7. Then serial subculture and neurosphere counting was repeated every 7 days, with 1 × 10^4^ NPCs being seeded at the beginning of every passage. MB was added into cell suspension at the final concentration of 5 μM at the time of plating cells. To assess NPC differentiation, neurosphere were triturated into single cells before plating onto poly-L-lysine-coated coverslips in Neurobasal medium supplemented with B27 free of FGF2 and EGF. MB (5 μM) was added at 24 h after plating.

### APOPTOSIS ASSAY

Neural progenitor cells were stained with propidium iodide (PI) and Annexin V-FITC (both from BD Biosciences, Franklin Lakes, NJ, USA) according to the manufacture’s instruction. Cells were analyzed on a BD LSRII flow cytometer.

### CELL PROLIFERATION AND CELL CYCLE ANALYSIS

For cell proliferation analysis, NPCs were stained with 5 μg/ml antibody against Ki67 (Abcam, Cambridge, MA, USA). For cell cycle analysis, NPCs were fixed with 70% ethanol and stained with PI (40 μg/ml, BD Biosciences, Franklin Lakes, NJ, USA) according to the manufacture’s instruction. DNA content was analyzed on a BD LSRII flow cytometer.

### IMMUNOCHEMISTRY

Cultured cells were fixed with 4% paraformaldehyde and stained with anti-MAP2 antibody (Santa Cruz, Dallas, TX, USA) followed by staining with Alexa Fluor 488-conjugated goat anti-rabbit IgG (Invitrogen, Grand Island, NY, USA). Fluorescent microscopy was conducted using an Axio Observer Z1 fluorescent microscope (Zeiss, Thornwood, NY, USA). On each slide, cells in five randomly selected microscopy fields were counted. For brain section staining, rats were anesthetized and perfused with neutral-buffered formalin. The brains were harvested and fixed in neutral-buffered formalin overnight before sinking in 30% sucrose. Frozen sections (20 μm) were prepared followed by staining with mouse anti-BrdU mAb and rabbit anti-Nestin antibody (both from Sigma-Aldrich, St. Louis, MO, USA). Sections were observed on a LSM 410 confocal microscope (Zeiss, Thornwood, NY, USA) to record the z-stack images to confirm colocalization of Nestin and BrdU-labeled cells in the SVZ. Images were analyzed with the LSM Image Browser software. BrdU- and Nestin-positive and double-labeled cells in the SVZ, along the lateral walls of the lateral ventricles (beginning at 1.18 mm anterior to bregma), were counted in four sections per animal, spaced 120 μm apart, Results were expressed as the average number of BrdU- and Nestin-positive cells in SVZ.

### WESTERN BLOT

Cells or tissues were lysed in RIPA buffer. The following primary antibodies were used to detect the proteins of interest: (1) cyclin antibodies (Cyclin Antibody Sampler Kit, Cell Signaling, Danvers, MA, USA); (2) mTOR mAb, 4E-BP1 mAb, p70 S6 kinase mAb, and GAPDH mAb (Cell Signaling, Danvers, MA,USA); (3) mouse GFAP mAb and rabbit DCX polyclonal antibody (Santa Cruz, Dallas, TX, USA). Protein bands were visualized and optical density was analyzed using a Biospectrum 500 imaging system (UVP, LLC, Upland, CA, USA).

### REAL-TIME RT-PCR

Total RNAs were extracted using TRIzol and were reversely transcribed to cDNAs using SuperScript®; III First-Strand Synthesis System according to the manufactures’ instructions. Real-time PCR was performed using Fast SYBR®; Green Master Mix on a 7300 Real-Time PCR System. Reagents and instruments were purchased from Invitrogen, Grand Island, NY, USA. Data was analyzed with 7300 system software. Primer sequences for each gene were shown as follows (Forward and reverse):

*Ccnd1:* 5′-ACTGACAACTCTATCCGCCC-3′;

             5′-TCTGTGCATGTTTGCGGATG-3′.

*Ccnd2:* 5′-AAAGAGACCATCCCGCTGAC-3′;

             5′-TGAAGTCGTGAGGGGTGACT-3′.

*Ccnd3:* 5′-GTACCGTCTGCCTGTTGCTG-3′;

             5′-CAGGAAGTCGTGCGCAATCA-3′.

*Ccne1:* 5′-CCGACCTCTCAGTCCGATCC-3′;

             5′-CCACGCACGCTGAATCATCA-3′.

*Ccne2:* 5′-CAGGCCTATATATTGAGTTGGCG-3′;

             5′-GGCTACTGCGTCTTGACATTC-3′.

*Ccna1:* 5′-CTGACCGTTCCAACCACCAA-3′;

             5′-CTGCTGCTACCAAGGAAGGAA-3′.

*Ccna2:* 5′-GCCAACTGCAAGGTAGAAAGC-3′;

             5′-GCTCCAGCAATAAGCGATGG-3′.

*Ccnb1:* 5′-TAGATGCAGACGATGGTGGTG-3′;

             5′-CAGTGACTTCACGACCCAGTA-3′.

*Ccnb2:* 5′-TAAGGCGAGCCTCAAAAGCC-3′;

             5′-ATGTCGTAGTCGACGAGGGT-3′.

*Ccnb3:* 5′-GGCACAATGCGAGAAGAACC-3′;

             5′-ACTCTGTTAGGGAAGGGGGA-3′.

*Map2:* 5′-AAGCGGAAAACCACAGCAAC-3′;

             5′-GGTCTTGGGAGGGAAGAACG-3′.

*Nestin:* 5′-GCACACCTCAAGATGTCCCT-3′;

             5′-GAACCTCGTCCAGGTGTCTG-3′.

*Dcx:* 5′-TCACAGCATCTCCACCCAAC-3′;

             5′-GTCCATTCATCCGTGACCCT-3′.

*Gfap:* 5′-GGAGAGGGACAATCTCACACAG-3′;

             5′-TTCCTCTCCAGATCCACACGA-3′.

*Gapdh:* 5′-GATGGTGAAGGTCGGTGTGA-3′;

             5′-TGAACTTGCCGTGGGTAGAG-3′.

### STATISTICAL ANALYSIS

Data were analyzed and results were presented as mean ± SD. Student’s *t* test or one-way ANOVA was used for comparison of mean between the groups, and *p* < 0.05 was considered statistically significant.

## RESULTS

### MB INHIBITS NPCs PROLIFERATION *IN VITRO*

**FIGURE 1:**
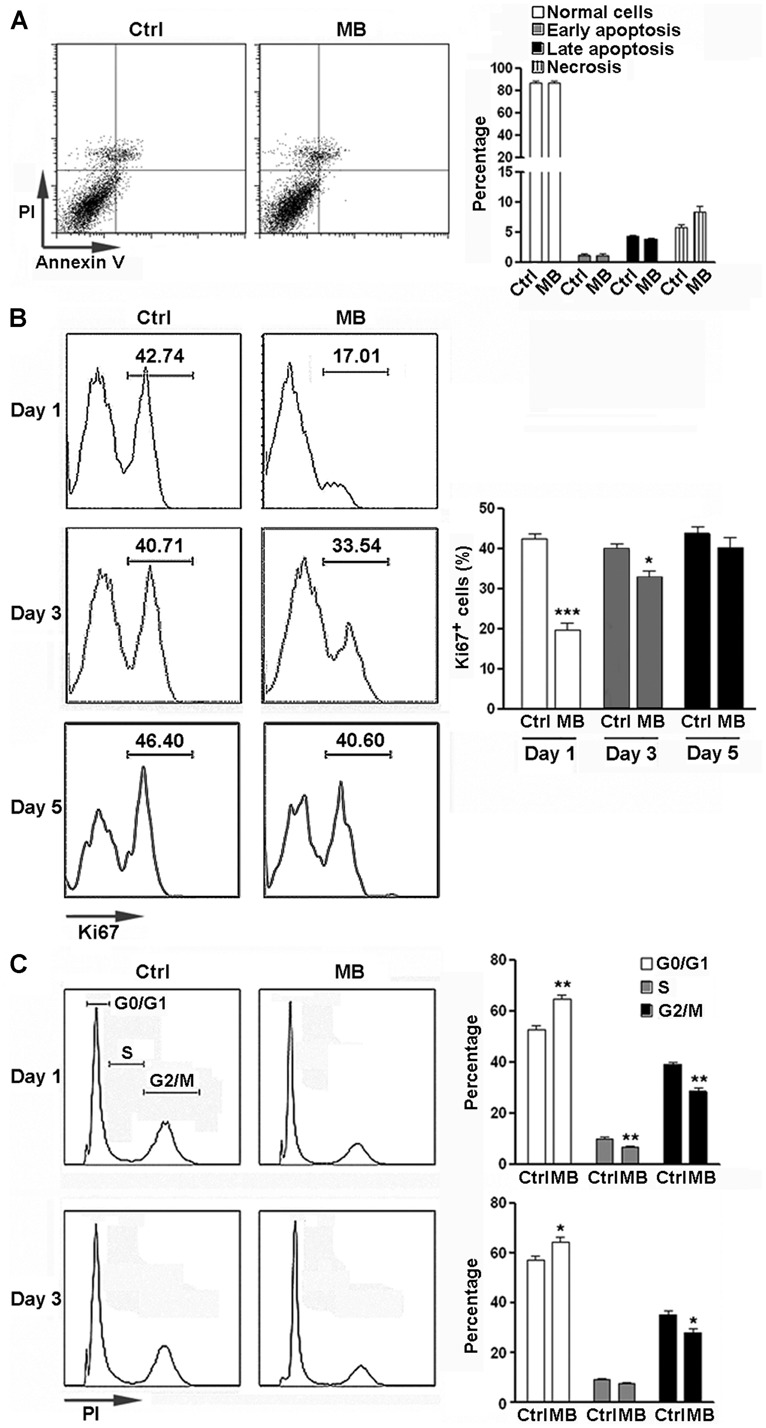
**MB inhibits NPC proliferation *in vitro*. (A)** NPC apoptosis after 6 day MB treatment. **Left:** representative dot plots. **Right:** statistical analysis. **(B)** Detection of NPC proliferation by Ki67 staining. **Left:** representative histograms. **Right:** statistical analysis. **(C)** Detection of NPC cell cycle by PI staining at 1 day and 3 days after MB treatment. **Left:** representative histograms. **Right:** statistical analysis. *N* = 6 in each group. Ctrl, control; MB, MB-treated cells. *p < 0.05; **p < 0.01; ***p < 0.001 compared with control group.

To determine the potential cytotoxicity of MB, NPCs were treated with 5 μM MB for 6 days and apoptosis was analyzed. No significant change in apoptosis and necrosis was observed after treatment (**Figure 1A**). We then determined the effect of MB on NPC proliferation by Ki67 staining. The proportion of Ki67^+^ cells were significantly reduced at day 1 and day 3 after MB treatment, compared with control group (**Figure 1B**). On day 5 after treatment, NPC proliferation returned to the control level (**Figure 1B**). Consistently, cell cycle analysis with PI staining indicated that MB treatment induced significant G0/G1 arrest in NPCs at day 1 and day 3 post-treatment (**Figure 1C**).

### MB DOWN-REGULATES CYCLIN EXPRESSION IN NPCs *IN VITRO*

**FIGURE 2:**
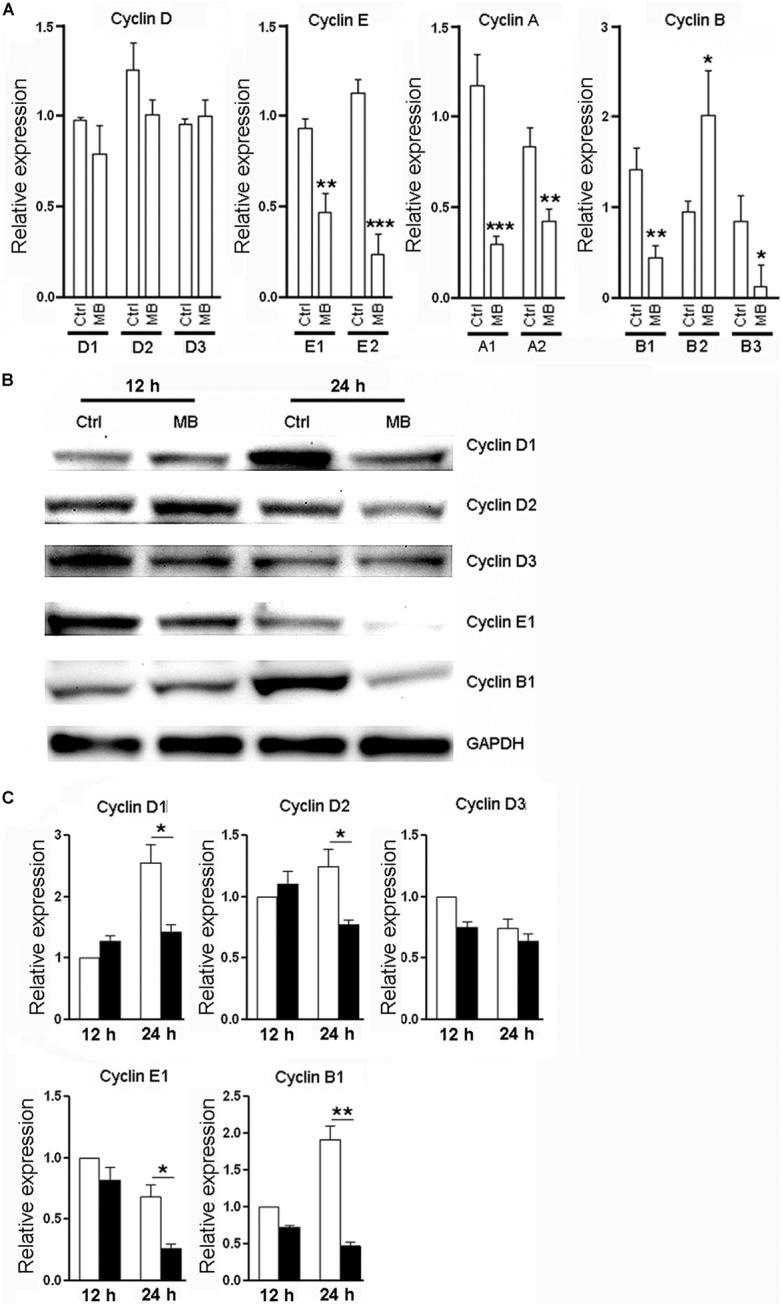
**MB down-regulates cyclin expression in NPCs. (A)** Detection of mRNA levels of cyclin (D,E,A,B) in NPCs at 24 h after MB treatment. **(B)** Western blot assay for cyclin expression in NPCs. This is a representative of three independent experiments. **(C)** Statistical analysis for cyclin protein levels. Expression level was normalized to each control group at 12 h after culture. *N* = 6 in each group. Ctrl, control; MB, MB-treated cells. *p < 0.05; **p < 0.01; ***p < 0.001 compared with control group.

Cyclins are a family of proteins that control cell progression through the cell cycle by activating cyclin-dependent kinases (Bloom and Cross, 2007; Lange and Calegari, 2010). Having observed the anti-proliferative effect of MB on NPCs, We then determined the effect of MB on cyclin expression in NPCs. Since **Figure 1B** showed that 1 day treatment had the most robust effect, we selected this time point to investigate. Consistent to its action on cell cycle, 24 h treatment with MB down-regulated various NPC cyclins at mRNA levels and/or protein levels. MB did not affect cyclin D1, D2, and D3 transcription, but significantly reduced the mRNA levels of cyclin E1, E2, A1, A2, B1, and B3, although there was an increase in cyclin B2 mRNA (**Figure 2A**). Western blot confirmed down-regulated expression of cyclin E1 and B1 after MB treatment (**Figures 2B,C**). In addition, protein levels of cyclin D1 and D2 were also down-regulated after MB treatment (**Figures 2B,C**), suggesting that MB inhibited translation or enhanced degradation of cyclin D1 and D2.

### MB ENHANCES NEUROSPHERE GENERATION IN SERIAL PASSAGE ASSAY

**FIGURE 3:**
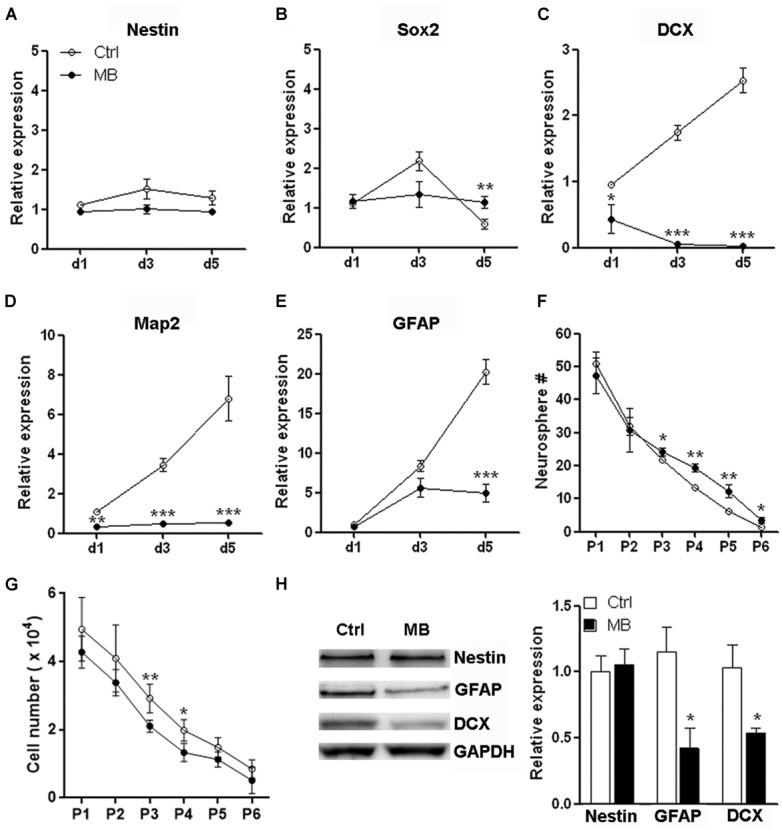
**MB maintains neurosphere formation capacity of NPCs. (A–E)** Detection of mRNA levels of stem cell, neural and glial markers in passage 1 (P1) NPCs. *N* = 3 in each group. **(F)** Serial passage analysis for neurosphere formation. *N* = 6 in each group. **(G)** Total cell number counting at the end of each passage. Note that at the beginning of each passage, only 1 × 10^4^ NPCs were seeded. **(H)** Western blot assay for Nestin, GFAP and DCX expression in P1 NPCs treated with MB for 6 days. **Left**, representative Western blot image. **Right**, statistical analysis for expression level of each protein. *p < 0.05; **p < 0.01; ***p < 0.001 compared with control group.

Neural progenitor cells are able to maintain their relatively undifferentiated state through self-renewal under both steady state and pathological conditions. Self-renewal is essential for stem cells to maintain stem cell pool within organs and tissues (He et al., 2009). To determine whether the MB-induced NPC quiescence favors neurosphere formation, we firstly tested mRNA levels of neural markers to determine NPC differentiation in the suspension culture. We found that NPCs progressively up-regulated expression of DCX, MAP2, and GFAP even in suspension culture with growth factors. Nestin and Sox2 expression were up-regulated from day 1 to day 3 but were reduced after day 3. MB maintained Nestin and Sox2 expression, while significantly inhibited expression of DCX, MAP2, and GFAP as compared with vehicle control (**Figures 3A–E**), suggesting MB restrained NPCs differentiation in suspension culture. We then performed serial passage assay to check the self-renewal capability of NPCs. We found that neurosphere number was decreased over serial passages in either group, while MB treatment generated more neurospheres from passage 3–6, suggesting that neurosphere generation was promoted by MB (**Figure 3F**). Consistent with less proliferation, MB decreased the total NPC number in each passage, with the most significant decrease displayed in passage 3 and 4 (**Figure 3G**). Western blot assay confirmed lower expression of DCX and GFAP in MB-treated cells (**Figure 3H**), suggesting that differentiation was indeed inhibited by MB.

### MB DOES NOT AFFECT COMMITTED NEURONAL DIFFERENTIATION FROM NPCs

**FIGURE 4:**
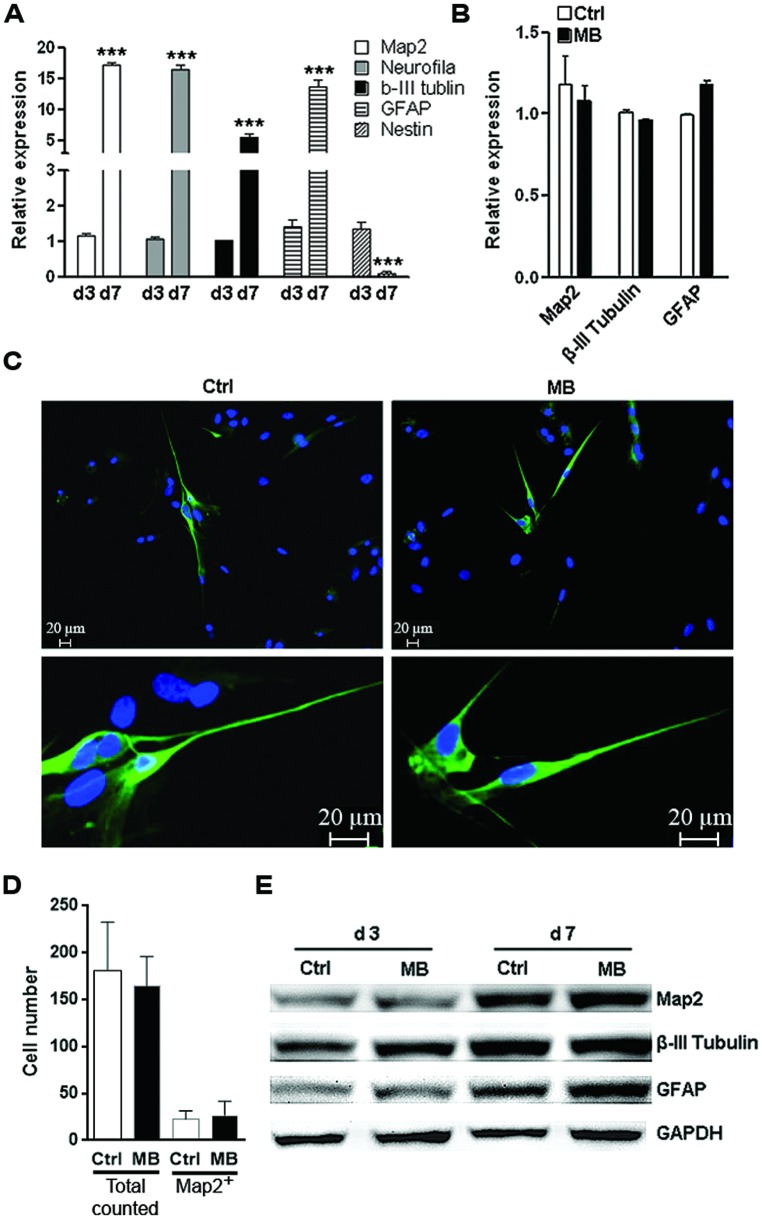
**MB does not impede committed neuronal differentiation. (A)** Q-RTPCR analysis of the expression of differentiation markers in differentiated NPCs at 1 and 7 days after growth factors withdraw. *N* = 3 per group. ***p < 0.001 compared with day-1 differentiated NPCs.** (B)** Q-RTPCR for expression of differentiation markers in differentiated NPCs in the presence or absence of MB. *N* = 3 per group.** (C)** Map2 staining for differentiated NPCs in the presence or absence of MB. **Upper**, 50×; **lower**, 200×. **(D)** Total cell number counting and Map2^+^ cell number in five microscopy fields (50×).** (E)** Western blot assay for expression of differentiation markers in differentiated NPCs in the presence or absence of MB. This is a representative of two independent experiments.

The other important function of NPCs is to differentiate into neurons and glia to sustain or repair the CNS structure. To test whether MB affected committed neuronal differentiation, we induced NPCs differentiation by withdrawing growth factors in adhesion culture for 7 days. Differentiation marker expressions were all robustly up-regulated while Nestin was significantly down-regulated on day 7, suggesting that induction of differentiation was successful (**Figure 4A**). MB did not significantly alter the expression of MAP2, tubulin β-III and GFAP at 7 days after growth factors withdraw (**Figure 4B**). Consistently, immunofluorescence staining showed comparable MAP2 expression in control and MB-treated cells, indicating that MB did not affect committed neuronal differentiation (**Figures 4C,D**). Western blot assay also demonstrated comparable level of each differentiation marker between control and MB-treated groups (**Figure 4E**). Thus, it seems that MB does not affect committed neuronal differentiation from NPCs.

### MB INHIBITS TRANSCRIPTION OF mTOR, p70S6K, AND 4EBP1

**FIGURE 5:**
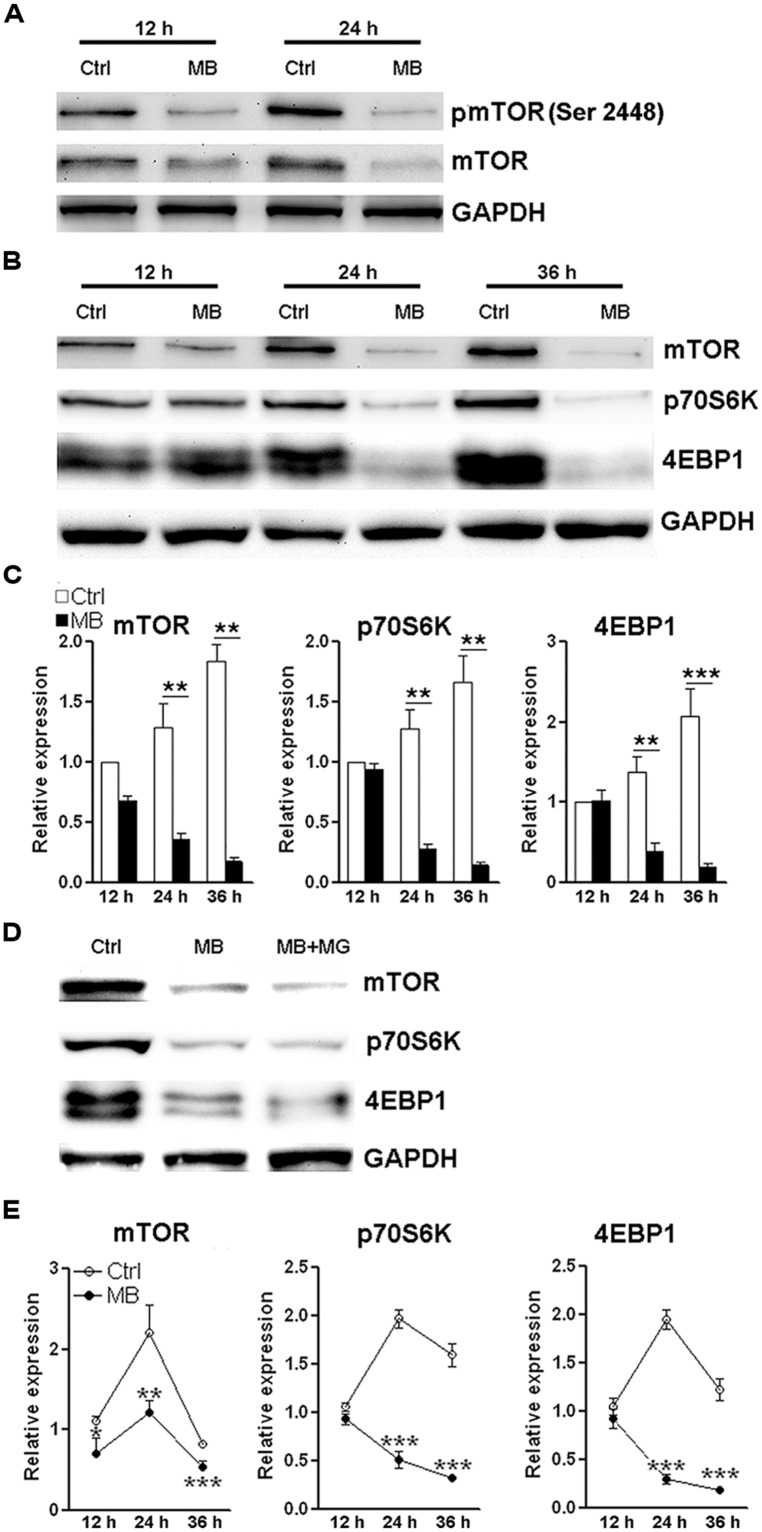
**MB down-regulates mTOR expression via decreasing mTOR transcription. (A)** Western blot assay of mTOR phosphorylation in NPCs. This is a representative of two independent experiments. **(B)** Western blot assay for the expression of mTOR signaling components in P1 NPCs. **(C)** Statistical analysis for protein levels of mTOR, p70S6K, and 4EBP1 after MB treatment. **(D)** Western blot assay for mTOR, p70S6K, and 4EBP1 in P1 NPCs treated with vehicle, MB, or MB plus proteasome inhibitor MG132. This is a representative of two independent experiments.** (E)** Q-RTPCR for mRNA levels of mTOR, p70S6K and 4EBP1 in P1 NPCs treated with MB. *N* = 3 in each group. *p < 0.05; **p < 0.01, ***p < 0.001 compared with control group.

The close relationship between mTOR signaling and cell cycle progression has been elucidated elsewhere (Fingar et al., 2004). Based on the above data showing MB impedes cyclin D translation, we speculated that MB down-regulates mTOR signaling to modulate NPC proliferation. So we first determined mTOR activating phosphorylation in MB-treated NPCs. We did observe decreased mTOR phosphorylation at Ser 2448 after MB treatment. In addition, we found the protein level of total mTOR was also reduced (**Figure 5A**). Thus, the decreased phosphorylated mTOR is likely due to decreased total mTOR protein. Then we determined the expression of two target proteins of mTOR -p70S6K and 4EBP1- in the mTORC1 signal pathway. We found that MB robustly down-regulated protein levels of both p70S6K and 4EBP1 (**Figures 5B,C**). To test whether MB enhances degradation of these proteins, NPCs were co-treated with proteasome inhibitor MG132. MG132 treatment did not increase protein levels of mTOR, p70S6K, and 4EBP1 in MB-treated NPCs, suggesting that protein degradation was not involved in MB-induced down-regulation of these proteins (**Figure 5D**). Real-time PCR revealed that MB significantly decreased mRNA level of mTOR, p70S6K and 4EBP1 (**Figure 5E**). Thus, it seems MB inhibits mTOR transcription and in turn decreases mTOR protein, leading to reduction of cyclin D translation and inhibition of cell cycle entry.

### MB ALLEVIATES NPC PROLIFERATION *IN VIVO*

**FIGURE 6:**
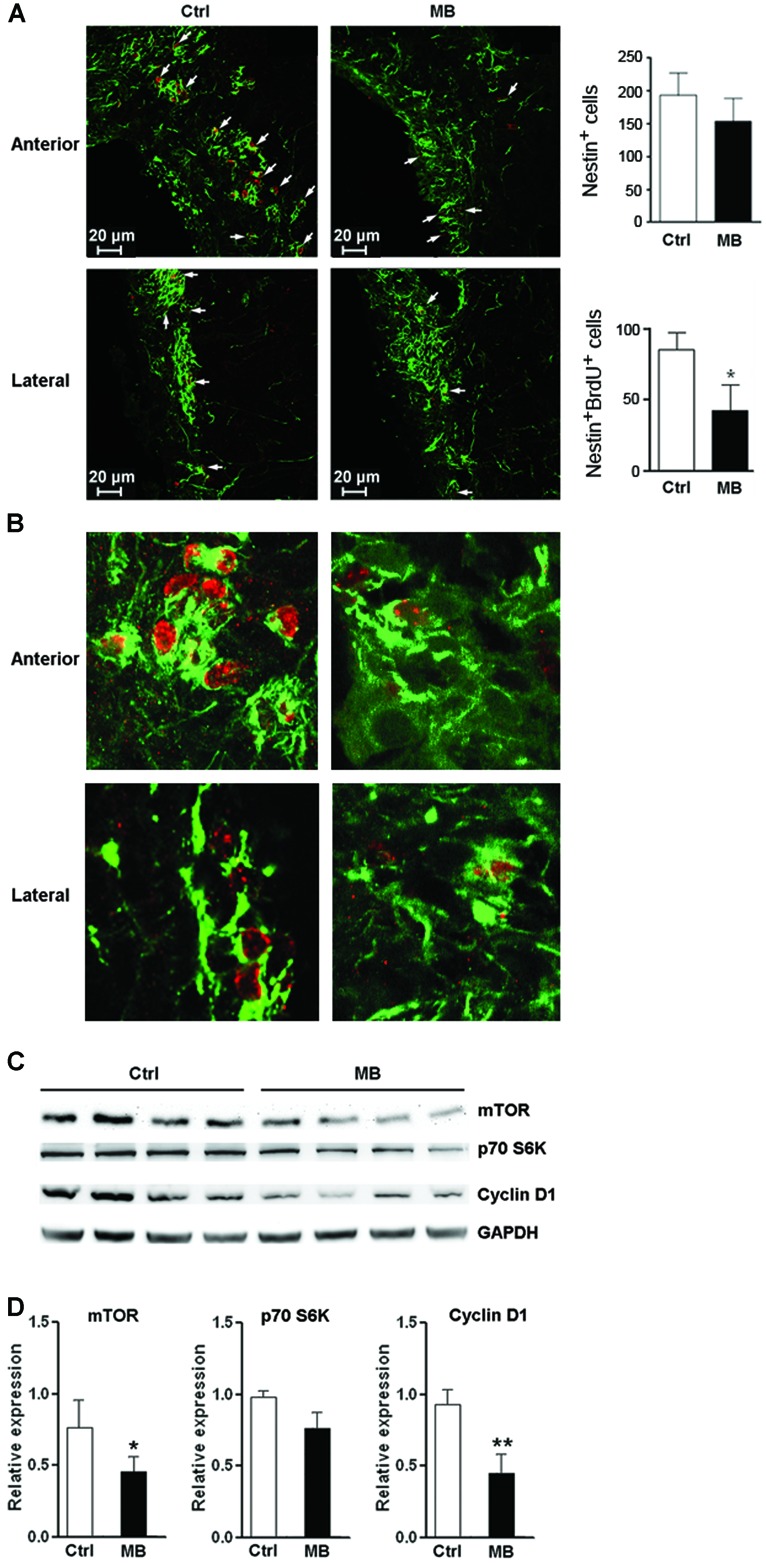
**MB restrains NPC proliferation *in vivo*. (A)** Detection of NPC proliferation with Nestin and BrdU staining. **Left**: representative confocal microscopy images (100×) of Nestin (green) and BrdU (red) at anterior and lateral part ventricular wall of SVZ in MB or vehicle treated rats. Arrows indicate Nestin^+^/BrdU^+^ cells. **Right**: quantification of Nestin^+^ cells and Nestin^+^/BrdU^+^ cells in the SVZ. **(B)** Representative confocal microscopy images (400×) of Nestin (green) and BrdU (red) at anterior and lateral part ventricular wall of SVZ.** (C)** Western blot assay for mTOR, p70S6K and cyclin D1 expression at SVZ in MB or vehicle-treated rats. **(D)** Statistical analysis for mTOR, p70S6K and cyclin D1 proteins at SVZ in MB or vehicle-treated rats. *p < 0.05; **p < 0.01 compared with control group.

To test whether MB also induces NPC quiescence *in vivo*, MB was intracerebroventricularly infused into adult rats for 7 days. Immunofluorescence staining of Nestin and BrdU demonstrated less Nestin^+^/BrdU^+^ cells at the SVZ in MB-treated rats, suggesting that MB inhibited NPC proliferation (**Figures 6A,B**). Consistent with our *in vitro* result, MB down-regulated mTOR and cyclin D1 expressions in the SVZ, while down-regulation of p70S6K was not as significant as that in *in vitro* assay (**Figures 6C,D**). Hence, we predicted that MB enhances the quiescence of NPCs by decreasing mTOR expression *in vivo*.

## DISCUSSION

Neural progenitor cells are captivating given their capacity to differentiate into multiple brain cell types yet maintain their undifferentiated state through self-renewal by which a stem cell divides asymmetrically or symmetrically to generate one or two daughter stem cells that have the developmental potential of the mother cell. The ability to self-renewal is essential for stem cells to expand their numbers during development and to maintain stem cell pool within adult tissues (He et al., 2009). The slow replenishment of degenerating cells with newly generated neuronal cells under physiological conditions *in vivo* has suggested that neural stem cells basically rest in a state of quiescence, which allows them to maintain a balance between the ability to undergo self-renewal and to differentiate without depleting the stem pool. The importance of stem cell quiescence for the long-term function of stem cells, including neural stem cells, has been demonstrated (Cheng et al., 2000; Kippin et al., 2005; Rera et al., 2011). Dysregulation and loss of quiescence often results in an imbalance in progenitor cell populations ultimately leading to stem cell depletion (Cheung and Rando, 2013). Thus, how to balance the NPCs proliferation/differentiation and quiescence to prevent premature senescence is of great interest in stem cell-related therapeutic strategies.

In this study we assessed the effect of MB on NPCs, focusing on two critical aspects of NPCs—quiescence and differentiation. Quiescence is an important property of adult stem cells for maintaining stem cell pool and preventing premature senescence. *In vitro* growth factor-driven serial passage assay continuously places neural stem cells under the proliferative stress so as to exhaust their neurosphere-forming capacity after several passages. MB slowed NPC proliferation evidenced by the reduction of Ki67 expression. MB treatment also induced NPC cell cycle arrest. As predicted, the inhibitory action of MB on NPCs was associated with an increase of neurosphere generation. Thus, our data indicated that MB induces NPCs into a relatively quiescent state. Noteworthily, MB treatment did not alter the expressions of mature neuronal markers when NPCs were cultured under differentiation condition. Thus, methylene treatment maintains NPC pool without affecting committed neuronal differentiation. The *in vivo* data proves that MB exerts similar inhibitory action on NPC proliferation at the SVZ. This is particularly beneficial for patients receiving MB, since their neuronal development is unlikely to be affected.

Cyclins are a family of proteins that control cell progression through the cell cycle by activating cyclin-dependent kinases (Bloom and Cross, 2007; Lange and Calegari, 2010). MB down regulates various cyclins in NPCs. The pattern of MB-induced changes of cyclin expression provided further clues for the mechanism by which MB modulates NPC proliferation. Our data demonstrated that MB did not alter mRNA level of cyclin D while decreasing its protein level, suggesting that MB down-regulated translation of cyclin D. Expressions of most other cyclins such as cyclin E, B, and A were also decreased by MB. MB-induced cyclin D decrease might hamper G0/G1 transition and subsequent cell cycle progression, resulting in cell cycle arrest, proliferation inhibition and quiescence. The action of MB to reserve quiescent NPC pool may provide a pharmacological intervention to increase the longevity of neural stem cells.

The well-known regulatory factor of cyclin D translation is mTOR, which controls cell growth and metabolism. mTOR signaling controls cell growth by regulating both translation (Vadlakonda et al., 2013) and transcription (Wander et al., 2011) of cell cycle factors. The close relationship between mTOR signaling and cell cycle progression has been elucidated (Fingar et al., 2004). Previous studies has indicated the involvement of mTOR signaling in the maintenance of human embryonic stem cells (Zhou et al., 2009), NPCs (Han et al., 2013), hematopoietic stem cells (Chen et al., 2008), and cancer stem cells (Matsubara et al., 2013). mTOR signaling is also important for both self-renewal and differentiation of neural stem cells (Magri et al., 2011). mTORC1 inhibition with rapamycin has been shown to improve stem cell function in the epidermis, in the hematopoietic system, and in the intestine (Castilho et al., 2009; Chen et al., 2009; Yilmaz et al., 2012). Recent studies indicated that MB regulates cell signal transduction such as mTOR (Congdon et al., 2012) and AMPK signaling (Xie et al., 2013). In our study, MB inhibited the expression of mTORC1 signaling components, including mTOR, 4EBP1, and p70 S6 kinase. The association between mTORC1 inhibition and cyclin D decrease poses the possibility that MB induces NPC quiescence through blocking mTORC1 pathway. However, further studies are in need to elucidate how MB affects expression of mTORC1 components. Our data is the first showing the transcriptional regulation of mTOR by MB. To our surprise, we can hardly find previous publications demonstrating transcriptional regulation of mTOR. Most research focused on the activation of mTOR via serine phosphorylation and complex formation. A recent study indicates that Sox2 suppress mTOR transcription, via binding at a region about 1.6 kbp upstream of the transcription start site of the mTOR gene (Wang et al., 2013). Another study showed that mTOR is overexpressed with loss of the Rb1 family pathway (El-Naggar et al., 2009). Whether MB utilizes these two signal pathways to regulate mTOR transcription needs further investigation.

In summary, the current study has provided the first *in vitro* and *in vivo* evidence that MB treatment promotes NPC quiescence through induction of cell cycle arrest. The inhibitory action of MB on NPC proliferation is accompanied by an inhibition of mTOR signaling. We speculate that MB treatment could maintain NPCs in a relative quiescence stage without affecting the differentiation potential. Our finding warrants future studies to determine the long term action of MB on NPCs and functional outcome in healthy and neurodegenerative disease models.

## AUTHOR CONTRIBUTIONS

Conducted experiments: Luokun Xie, Gourav R. Choudhury, Jixian Wang, Yong Park, and Ran Liu.

Data analysis: Luokun Xie.

Research design: Luokun Xie, Kunlin Jin, and Shao-Hua Yang.

Wrote or contributed to the writing of the manuscript: Luokun Xie, Chun-Li Zhang, Fang Yuan, Thomas Yorio, and Shao-Hua Yang.

## Conflict of Interest Statement

The authors declare that the research was conducted in the absence of any commercial or financial relationships that could be construed as a potential conflict of interest.

## ABBREVIATIONS

CNS: central nervous system
DCX: doublecortin
GFAP: glial fibrillary acidic protein
MAP2: microtubule-associated protein 2
MB: methylene blue
NPCs: neural progenitor cells
SVZ: subventricular zone.
